# Control of a *Mycoplasma pneumoniae* Outbreak in an Institutional Setting Using Azithromycin Prophylaxis

**DOI:** 10.3389/fpubh.2017.00366

**Published:** 2018-01-22

**Authors:** Michael Gdalevich, Eric J. Haas, Larisa Dukhan, Manuel Katz, Victoria Zelenski, Jacob Moran-Gilad

**Affiliations:** ^1^Israeli Ministry of Health, Southern District, Beer-Sheva, Israel; ^2^Faculty of Health Sciences, Department of Health Systems Management, Ben Gurion University of the Negev, Beer-Sheva, Israel; ^3^Division of Pediatrics, Faculty of Health Sciences, Ben Gurion University of the Negev, Beer-Sheva, Israel

**Keywords:** *Mycoplasma pneumoniae*, outbreak, residential, azithromycin, prophylaxis, developmental disabilities

## Abstract

**Background:**

*Mycoplasma pneumoniae* is a major cause of respiratory infection of varying severity. Outbreaks of *M. pneumoniae* infection commonly occur in closed or semi-closed communities and settings. The control of such outbreaks is challenging, owing to delayed detection, long incubation period, and paucity of infection control guidelines.

**Methods:**

Between May and July 2015, a residential facility for adults with developmental disabilities in Southern Israel witnessed an outbreak of acute respiratory infection, subsequently diagnosed as associated with *M. pneumoniae*. All relevant data were collected as a part of a formal outbreak investigation. Strict infection control procedures were implemented, and azithromycin prophylaxis was provided to all residents.

**Results:**

Out of 215 residents, there were 29 suspected cases, 23 of which were confirmed as *M. pneumoniae* infection by serology or nucleic acid testing, for an attack rate of 11%. There were no cases of severe or fatal illness. An infection control strategy, including implementation of strict case isolation, enforcement of hygiene measures, a high index of suspicion for case detection, and use of azithromycin prophylaxis for all residents, led to rapid cessation of the outbreak.

**Discussion:**

The use of azithromycin prophylaxis may be worthwhile in closed institutional settings in which *M. pneumoniae* infections are documented. The dynamics of this outbreak suggest that if spread between wards is anticipated, expanding prophylaxis beyond immediate contacts of affected individuals should be considered.

## Introduction

*Mycoplasma pneumoniae* is a common cause of upper respiratory tract infection and community-acquired pneumonia (1). This smallest self-replicating organism capable of cell-free existence is spread both by direct contact between an infected person and a susceptible person and by droplets expelled when an infected person sneezes, coughs, or talks (2). The incubation period varies, averaging about 2–3 weeks (range 1–4 weeks). Incidence is higher in late fall or winter, although reported outbreaks seem more common in late summer and autumn.

In community settings, this infection easily spreads between family members, and recurrence is common as immunity is short-lived (3). Outbreaks of *M. pneumoniae* have been described in military recruits (4), hospitals (5), and residential or long-term care facilities (6), as well as in the community (7). The combination of high infectivity, relatively long incubation period and short-lived immunity may lead to prolonged outbreaks, lasting months and even years (8, 9). We describe herein an outbreak of *M. pneumoniae* affecting institutionalized adults with developmental disabilities and the use of antimicrobial prophylaxis as part of the public health intervention aimed to control the outbreak.

## Materials and Methods

The outbreak setting is a residential facility for adults with developmental disabilities located in Dimona, a small town (population: 33,500) in Southern Israel. There are 215 permanent residents in the “Telalim” facility, accommodated in 10 separate wards, grouped by the severity of mental impairment and resulting mobility, dependence, and impact on daily living activities. Each room houses two to three residents, and every two rooms share a bathroom. There is little or no mixing of residents between wards, but staff members frequently rotate as needed.

In May 2015, there were two cases of acute febrile respiratory illness in two different wards. In June, there were 13 additional cases, including 10 from the same wards affected in May and 3 from three other wards. The District Health Office Epidemiology Unit was informed of the occurrence of acute febrile respiratory illness on July 8, 2015. Immediate concurrent infection prevention and control (IPC) measures were instituted in the entire facility, including standard infection control precautions, strict respiratory droplet precautions (staff were required to wear surgical face masks), cohorting of ill patients, and education of the staff members regarding case detection and infection control. These preventive measures were monitored by the DHO team and breaches in infection control were rectified.

The DHO Epidemiology Unit team conducted an epidemiologic investigation in order to better characterize the outbreak, establish an epidemic curve and outbreak dynamics and devise prevention and control measures. All residents of Telalim, presenting between May 1 and July 31, with fever ≥38.0°C and/or respiratory symptoms (cough, rhinorrhea, sore throat), were considered as suspected cases of *M. pneumoniae* infection. Confirmed cases were defined by laboratory evidence for *M. pneumoniae* infection. The latter consisted of serological evidence for infection (10) or positive nucleic acid testing of nasopharyngeal samples performed during admission to the regional hospital (Soroka University Medical Center) Emergency or Inpatient wards. Probable cases had an illness consistent with *M. pneumoniae* infection unexplained by an alternative diagnosis and no laboratory confirmation.

Public health physicians, nurses, and environmental health specialists performed onsite visits at the institution and provided recommendations for case identification, isolation with droplet precautions, and antimicrobial prophylaxis. Due to antimicrobial stewardship considerations, prophylaxis was recommended only for the wards in which there were cases; instructions were given to expand prophylaxis to additional wards should new cases arise. Prophylaxis was given to all ward residents, on top of IPC, using azithromycin, once daily, 500 mg on the first day and 250 mg for four additional days. The same regimen was recommended to all staff members.

Basic demographic and clinical information were recorded for all cases using Microsoft Excel and an epidemic curve was constructed to characterize the spread of disease over time. The outbreak investigation and response were exempt from ethical approval as part of routine public health surveillance and intervention.

## Results

**Figure 1:**
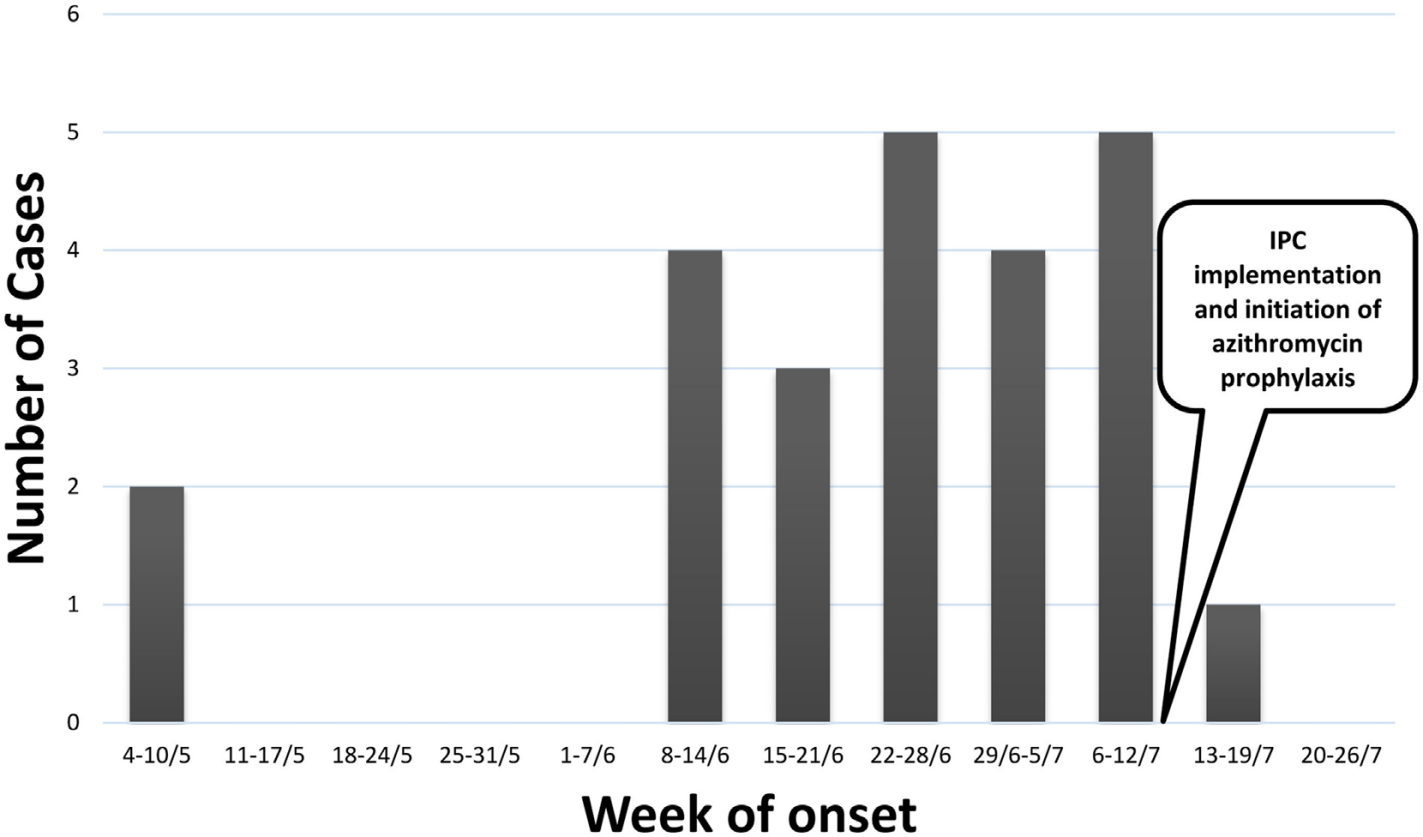
Epidemic curve of cases meeting the case definition for *Mycoplasma pneumoniae* infection (*n* = 24).

During the management of the outbreak, 29 patients had a suspected *M. pneumoniae* infection. Of those, 24 eventually met the case definition, either probable (*n* = 1) or confirmed (*n* = 23). Patients’ characteristics are presented in Table 1. Sixteen patients were referred to the hospital, of whom 4 were discharged from the emergency department and 12 admitted to medical wards. The remaining eight patients were treated at the institution’s clinic. Figure 1 shows the distribution of cases by week of onset. Disease severity was mild to moderate and there were no intensive care unit admissions or fatalities.

**Table 1:** Characteristics of outbreak cases.

S. No.	Start of symptoms	Gender	Age	Hospitalization	Positive serology or/and PCR	Ward	Case definition
1	04/05/2015	M	64	–	Serology+	1	Confirmed
2	10/05/2015	F	70	–	Serology+	1	Confirmed
3	09/06/2015	F	69	–	Serology+	2	Confirmed
4	10/06/2015	M	51	SUMC^a^	Serology+	1	Confirmed
5	10/06/2015	M	54	ED	Serology+	2	Confirmed
6	14/06/2015	F	65	–	Serology+	2	Confirmed
7	18/06/2015	M	53	ED	Serology+	1	Confirmed
8	20/06/2015	M	32	ED	Serology+	3	Confirmed
9	21/06/2015	M	58	–	Serology+	2	Confirmed
10	22/06/2015	F	53	–	Serology+	2	Confirmed
11	24/06/2015	F	55	–	Serology+	2	Confirmed
12	25/06/2015	M	44	SUMC	Serology+	3	Confirmed
13	26/06/2015	M	53	SUMC	Serology+	4	Confirmed
14	29/06/2015	M	36	SUMC	Serology+PCR+	3	Confirmed
15	02/07/2015	M	45	SUMC	Serology+	3	Confirmed
16	02/07/2015	M	40	–	Serology+	3	Confirmed
17	03/07/2015	M	43	SUMC	Serology+	3	Confirmed
18	07/07/2015	M	47	SUMC	Serology+PCR+	3	Confirmed
19	08/07/2015	M	62	SUMC	Serology+PCR+	5	Confirmed
20	10/07/2015	F	68	SUMC	Serology+PCR+	6	Confirmed
21	10/07/2015	M	56	ED	Serology+	5	Confirmed
22	11/07/2015	F	68	SUMC	Serology−	7	Probable
23	15/07/2015	F	40	SUMC	Serology+	3	Confirmed
24	15/07/2015	M	75	SUMC	Serology−	7	–
25	18/07/2015	M	61	–	Serology−	8	–
26	18/07/2015	F	66	SUMC	Serology+	2	Confirmed
27	26/07/2015	F	31	SUMC	Serology−	9	–
28	27/07/2015	M	69	SUMC	Serology−	9	–
29	28/07/2015	M	39	SUMC	Serology−	9	–

*^a^Soroka University Medical Center in Beer Sheba, Israel*.

All patients with *M. pneumoniae* infection received appropriate antimicrobial treatment, consisting of macrolides or fluoroquinolones. Of 29 suspected cases, 23 were confirmed cases, including 19 confirmed by serology and 4 by nucleic acid testing. Two suspected cases were consequently ruled out by serology at 2, 4, and 6 weeks after onset of illness. One of those cases was also negative by nucleic acid testing during initial hospitalization. Another three suspected cases eventually had alternative diagnoses.

The time interval between cases in the same ward appeared to be variable, consistent with what is known about the incubation period of *M. pneumoniae* (10). Antimicrobial prophylaxis was initiated starting July 13, 2015, in all wards, where suspected cases were present. Additional wards received prophylaxis, when newly suspected cases were identified. Ultimately, between July 13 and 18, 2015, all residents of the institution received prophylaxis and all staff members (*n* = 50) were referred to their primary care physician to receive prophylaxis. There were two confirmed cases after initiation of prophylaxis, 1 case diagnosed 2 days after initiation (i.e., the patient became symptomatic while receiving antibiotic prophylaxis) and 1 case in the remaining ward that had not yet received prophylaxis until July 18. This ward is a secluded facility for the most debilitated residents, all of whom are bedridden. This raised the possibility that staff transmission accounted for some of the spread as opposed to communicability between residents.

## Discussion

This report describes an outbreak of *M. pneumoniae* infection among residents of a facility for adults with developmental disabilities. We believe that in this outbreak of Mycoplasmal respiratory illness, administration of antimicrobial prophylaxis combined with strict environmental and personal hygiene enforcement contributed to the rapid, successful resolution. These findings raise several important issues.

*Mycoplasma* infections can present with varying levels of severity, ranging from upper respiratory symptoms with no fever to life-threatening pneumonia requiring intensive care support (11). Persons with Down syndrome, a common cause of mental retardation, seem to be at increased risk for *Mycoplasma* infection and severe disease, possibly due to immunological dysfunction (12). In general, mental retardation may predispose to lower respiratory tract infection resulting from impaired clearance of secretions, immobility, and additional comorbidities (13). For these reasons, we used a broad category of respiratory illness for the suspecting *M. pneumoniae* infection prior to laboratory confirmation.

Infection control is expected to be a challenge in this population, for whom the ability to comply with basic hygienic measures is low and personal, as well as group activities, involving close contact between residents and staff are common. The institution was given strict instructions to keep suspected cases at sufficient distance from other residents to prevent ongoing spread and to optimize environmental and personal hygiene management throughout the whole institution. These recommendations were fully implemented under close supervision by the DHO team.

Although no cases were identified among institution’s staff members, we viewed their rotation between different wards as an important potential contributor to the propagation of the outbreak. Given that one of the affected wards was a closed facility for non-ambulatory patients, where the possibility of mixing between residents and entrance of residents from other wards were non-existent, it is likely that asymptomatic or mildly symptomatic staff members may have contributed to spread within wards and between them.

The role of antimicrobial prophylaxis in a setting, such as the one under investigation, is not clear. The Control of Communicable Diseases Manual (20th edition) states specifically for *Mycoplasma* pneumonia, under “epidemic measures,” that “no reliably effective measures for control are available, although antimicrobial prophylaxis has been used in some institutional outbreak settings” and the Red Book (29th edition) is also equivocal on that point and targets mainly close contacts in institutional or family settings. Furthermore, the latest medical literature is scarce and controversial. A few studies have demonstrated the benefit of antimicrobial prophylaxis in closed, residential settings (14), as well as for health-care workers (15). A trial in military recruits also demonstrated the efficacy of azithromycin in reducing *Mycoplasma* carriage (16). However, a report on a large outbreak in USA, with a significant case-fatality rate did not mention any attempt to reduce spread with chemoprophylaxis (17).

The decision concerning antimicrobial prophylaxis is further complicated by the possibility of macrolide resistance. *M. pneumoniae* strains resistant to macrolides have emerged worldwide and have been spreading across continents. Recent studies have documented macrolide resistance in Israel in over 20% of cases (18). However, these data represent only a single region in the Jerusalem District, and local data from Southern Israel where the outbreak occurred are lacking. Other considerations in determining the role of prophylaxis include drug safety (allergy and adverse reactions), antimicrobial selective pressure as well as costs.

Our experience suggests that initiating antimicrobial prophylaxis for each ward that experienced at least 1 case curtailed further spread of disease. We adopted a conservative approach, mindful of antimicrobial stewardship and limited prophylaxis only to patients in implicated wards. It could be argued, that given the rotation of staff members on multiple wards, and most of the wards being open-door facilities, that treatment could have been recommended, from the onset of the event, for all residents and staff members on all wards simultaneously. In this outbreak, occurrence of additional cases led eventually to administering prophylaxis to the entire institution, supporting the latter approach. Thus, several epidemiological factors should influence such decision, whether to administer prophylaxis sequentially or in-full at the beginning of intervention. The dynamics of the outbreak, the timing of reporting and the projected course of the outbreak, based on risk assessment, are among these factors.

It is plausible that the earlier the intervention, the more effective will be the role of prophylaxis. In the outbreak reported here, the attack rate was over 10% at the point of intervention and, therefore, it is reasonable to believe that the pool of susceptibles was yet unexhausted. In light of the abrupt cessation of morbidity following intervention, it may be argued that prophylaxis has contributed to the control of the outbreak. Additional studies reporting on the use of prophylaxis in similar epidemiological settings are warranted.

## Conclusion

We describe an outbreak of *M. penumoniae* respiratory illness in a residential facility for adults with developmental disabilities. Implementation of infection control measures coupled with administration of antimicrobial prophylaxis successfully halted the spread of the disease. This suggests that a liberal use of antimicrobial prophylaxis can be considered in such an epidemiological context.

## Ethics Statement

The study was exempt from IRB approval as it was an outbreak investigation performed by the State authorities under public health national law. All data were obtained as part of the investigation and no additional research or experimentation has taken place.

## Author Contributions

MG, EH, and JMG designed the report and drafted the paper; LD, MK, and VZ were responsible for data acquisition; all authors have contributed to data analysis and interpretation and provided critical comments.

## Conflict of Interest Statement

The authors declare that the research was conducted in the absence of any commercial or financial relationships that could be construed as a potential conflict of interest.
